# PAPE Effect in Female Footballers: Analyzing the Benefits of Different Flywheel Protocols

**DOI:** 10.3390/sports13110370

**Published:** 2025-10-22

**Authors:** Pablo Asencio, José Luis Hernández-Davó, Marco Beato, Rafael Sabido

**Affiliations:** 1 Sport Research Centre, Department of Sport Sciences, Miguel Hernández University, 03202 Elche, Spain; rsabido@umh.es; 2 Faculty of Health Sciences, Isabel I University, 09003 Burgos, Spain; jlhdez43@gmail.com; 3 School of Allied Health Sciences, University of Suffolk, Ipswich IP4 1QJ, UK; m.beato@uos.ac.uk

**Keywords:** half-squat, lunge, post-activation performance enhancement, PAPE, female footballers, flywheel training

## Abstract

Post-activation performance enhancement (PAPE) is an acute performance increase in voluntary exercises induced by a conditioning activity. Due to the scarcity of evidence about the effectiveness of distinct protocols, the aim of this study was to compare the effects of two different flywheel PAPE protocols (half-squat and lunge exercises) on vertical and horizontal jump performance, as well as change-of-direction ability in female amateur footballers (*n* = 21). Each protocol consisted of 3 sets of 6 repetitions for the half-squat protocol or 10 repetitions for the lunge protocol, with two minutes of passive rest, performed with a conical pulley. Both protocols were followed by rests of two, eight, and twelve minutes for repeated countermovement jump (CMJ), triple hop, and change-of-direction test (modified T-505) testing. The fixed-effect model 2-ways-repeated measures ANOVA showed that there was no significant interaction between time and exercises performed (*p* > 0.05). There was no significant relationship between exercise specificity and performance in sport-specific tasks. Our results suggest that, within this population, neither flywheel protocol provided measurable PAPE benefits across varied time windows. The findings underscore the importance of strength levels in achieving PAPE benefits and question the specificity of PAPE protocols to targeted sport performance outcomes.

## 1. Introduction

Post-activation potentiation (PAP) and post-activation performance enhancement (PAPE) have been widely debated in performance science [1,2,3]. While PAP traditionally refers to increases in twitch force during electrically evoked contractions, PAPE describes acute improvements in voluntary performance tasks induced by a conditioning activity (CA) [3,4,5]. This distinction underlines the relevance of PAPE as a practical phenomenon for sport performance. Warm-up activities usually aim to increase the athlete’s body temperature and cardiometabolic and neuromuscular responses; PAPE can be considered one of these responses [1,2]. Several physiological mechanisms, such as increments in muscle temperature, muscle water content, and muscle activation, underlie the PAPE phenomenon [6]. The training level is a key modulating factor for the PAPE response [7], and all of these physiological mechanisms have been shown to occur in various tasks following specific warm-ups that activate the same muscle groups involved in testing activities, such as jumping [4], sprinting [8], change-of-direction (COD) ability [9], and upper-body ballistic exercises [10,11].

Due to their specific characteristics (e.g., higher force production, lower energy expenditure, specific motor unit recruitment, greater cortical activity or kinetic efficiency), eccentric actions may be optimal to enhance PAPE responses in comparison with isometric and concentric actions [6,12]. One way to focus in the eccentric phase of the movements is the use of flywheel resistance training, whose use has been increased markedly in recent years. Among the applicability of flywheel devices, their use in PAPE protocols has recently been highlighted [6,12]. The main reason to use flywheel resistance exercises to obtain some PAPE effects is associated with the muscle lengthening and concentrated braking action at the end of the eccentric phase, producing eccentric overload (EOL) phenomenon [6]. To obtain a PAPE effect using flywheel devices, it is very important that the exercises exhibit the proper technique to achieve EOL [13] and be challenging enough to enhance performance but not so demanding as to cause acute neuromuscular fatigue [14]. Most studies using flywheel exercises showed that the use of 3–4 sets of six repetitions of squat exercise can elicit PAPE in the minutes following the exercise, specifically between 3–9 min [15,16,17]. Despite some training variables (i.e., volume or intensity) being studied, there is not enough evidence about the influence of different training variables (i.e., force vector and type of exercise) in PAPE protocols, since the inertias used in these studies were highly variable (i.e., 0.03–0.11 kg·m^2^) [18].

Moreover, to maximize sport performance, the ability to perform both horizontal and vertical displacements is a crucial factor, particularly in multidirectional sports like football, where sprinting, jumping, and COD are decisive actions [19]. Besides the fact that PAPE could optimize specific football tasks, it remains unclear whether different types of conditioning activities (e.g., horizontal- or vertical-directed) may optimize PAPE effects (e.g., COD, vertical, and horizontal jumping) [15]. Previous studies have reported improvements in vertical performance variables following vertically directed flywheel exercises. For example, Beato et al. [15] observed increases in countermovement jump (CMJ) height at 3 and 7 min and in standing long jump distance at 3 and 7 min after a flywheel squat exercise. Similar improvements have also been reported [9] for other types of jumps, such as horizontal jumps, as well as for the “modified T-505” change-of-direction test (T-505) in both the dominant and non-dominant legs, 4 min after vertically, horizontally, and guided-directed tasks. Other authors [20] found significant improvements in vertical (CMJ) and horizontal (10–30 m sprint) performances following a vertically directed conditioning activity (flywheel squat) using medium (ML) and high (P) inertia loads, with CMJ enhancements observed immediately with P and peaking at 4 min post-intervention with both ML and P; sprint performance improved with ML at 4 min and was maintained at 8 min. In contrast, McErlain-Naylor & Beato [21] reported that the use of a vertically directed flywheel exercise (i.e., squat) did not improve horizontal jump performance. Despite these findings and the ongoing debate on the role of exercise specificity in PAPE, no previous studies have examined the influence of unilateral conditioning activities such as the flywheel lunge, particularly on horizontal and unilateral tasks in women’s football. It remains uncertain how the effectiveness of such protocols may vary across different recovery intervals (i.e., 2, 8 and 12 min), representing a critical gap addressed in the present investigation.

The aim of this study was to compare the effects of two different flywheel PAPE protocols (half-squat and lunge exercises) on vertical and horizontal jump performance, as well as change-of-direction ability in female amateur footballers, across different recovery time intervals (2, 8, and 12 min). We hypothesized that horizontal and unilateral flywheel exercises would maximize PAPE effects on horizontal and unilateral tasks (i.e., horizontal jump and COD ability) due to the force vector direction and the specificity of horizontal actions, while vertical flywheel exercise would induce a greater PAPE effect on vertical and bilateral jumping performance (i.e., CMJ), with potential differences across the tested recovery intervals.

## 2. Materials and Methods

### 2.1. Participants and Sample Size Estimation

A power analysis was performed (G*Power version 3.1.9.7, Heinrich Heine University Düsseldorf, Germany). This analysis showed that 18 participants were the minimum sample size to provide a statistical power of 80%, using an α of 5%, with an effect size of 0.3 and correlation (r = 0.6) between repeated tests. Twenty-one (*n* = 21) semi-professional female footballers took part in this study (age 20.73 ± 3.91 years; height 1.65 ± 0.06 m; body weight 58.54 ± 5.74 kg; one-repetition maximum (1-RM) squat 78.67 ± 11.30 kg; and ratio 1-RM/BW 1.22 ± 0.42). Besides the fact that they were semi-professional female footballers, according to the criteria by Meier et al. [22] (1 RM/BW = 1.40), they were not expert in squat exercise. Criteria for participation were the absence of injury or illness, verified through the Physical Activity Readiness Questionnaire, and regular participation in football training (at least two sessions per week during the last six months). Exclusion criteria included any musculoskeletal disorder, recent surgery, or medical condition that could limit high-intensity exercise performance, as well as failure to complete all experimental sessions. Participants and legal guardians were informed about risks and benefits of the current procedures and signed an informed consent form. The Ethics Committee of the University of Suffolk (Ipswich, UK) approved this study. The study was conducted in accordance with the Declaration of Helsinki and approved by the Ethics Committee of Universidad Miguel Hernández (Elche, Spain) on 6 March 2023 (Code: ADH.DES.RSS.PAV.23).

### 2.2. Study Design

**Figure 1:**
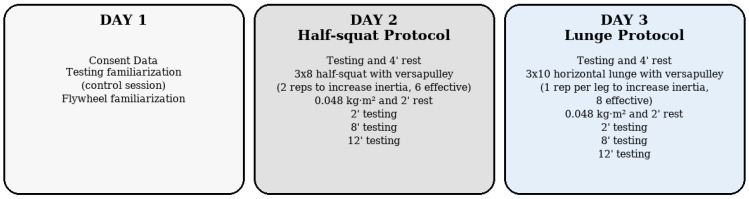
General scheme of the procedure. The protocol’s order of execution (Protocol A or B) was randomized and counterbalanced across participants.

This study had a randomized crossover design to check the effects of two different flywheel-based PAPE protocols (horizontal-unilateral vs. vertical-bilateral) during sport-specific tasks (see Figure 1). The order of execution was randomized across participants using a computer-generated random sequence in JASP software (version 0.19.3). The experiment took place on three days separated by one week, and each session was performed three days before competition (match day—3, 72 h before the next match).

The first day was used to collect baseline data and to familiarize participants with flywheel protocols and the performance test: CMJ, triple-hop test, and T-505. All participants were already familiar with the flywheel test procedures and training. Following that, the participants performed one specific PAPE protocol (half-squat and lunge exercises) for each day after a standardized warm-up. Performance assessments were conducted exactly four minutes after the conditioning exercise in a randomized order.

### 2.3. Procedures

A stadiometer (Seca 286dp; Seca, Hamburg, Germany) was used to record body mass and height. After having their anthropometric and descriptive data taken, participants performed a standardized warm-up: 5 min of cycling at a constant power on an ergometer (Sport Excalibur lode, Groningen, The Netherlands) followed by full-body mobility for both PAPE conditions as a warm-up [15]. Mobility consisted of dynamic movements for the main joints for the protocol (hip, knee, and ankle), one set of core stability exercises, ten repetitions of multiarticular movements imitating the main PAPE protocol exercise (e.g., half-squat and lunges), and two submaximal attempts separated by 30 s of different tests (CMJ, triple hop, and T-505). Coefficient of variation (CV) and intraclass correlation coefficient (ICC) were calculated to assess the variability and reliability of measurements.

Countermovement Jump. A contact platform (Chronojump v2.5.2; Boscosystem SL, Barcelona, Spain) was used to evaluate CMJ performance. Participants were instructed to perform their maximal jump height during three attempts separated by 30 s, with hands on their hips and being able to execute the jump descending to their preferred depth. The mean of three attempts [23] was used for the analysis. The CMJ CV and ICC values were 11.91% and 0.77, respectively.

Triple-Hop Test. Two attempts for the preferred leg of triple hops (arm swing allowed) were recorded in each session [24] and used for the analysis. Participants started with their toes positioned close behind the start line and hopped as far as possible for three hops, landing and holding their position for three seconds on the final hop after they were instructed to “stick” [24]. The CV of the test was 7.43%, while the ICC value was 0.73.

Change of Direction. Change of direction was tested at five meters of distance, consisting of two attempts of running and passing a line positioned 5 m away and performing a change of direction with the dominant leg turning 180° unilaterally (modified T-505), as typical in many sports [25]. Two timing gates (Microgate, Bolzano, Italy) were positioned at the start and end locations of the COD task in a standardized manner. Tests started on the “Go” command from a standing position, with the front foot 0.2 m from the photocell beam [26]. The COD task had a CV and ICC of 4.47% and 0.48.

### 2.4. Intervention

**Figure 2:**
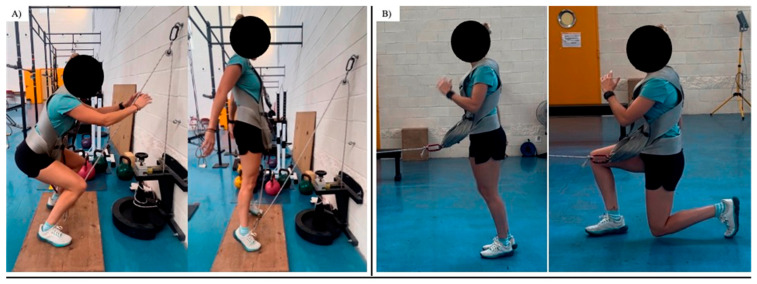
Half-squat (**A**) and lunge exercises (**B**).

This research used cross-over counterbalanced protocol. The measurements were conducted across two distinct sessions, preceded by one familiarization session. In these preliminary sessions, athletes warmed up and later performed six attempts for each test, with 30′′ of rest between attempts. The athletes were then instructed in the proper execution of each testing and exercise protocol (see Figure 2) in order to maximize the EOL. The PAPE protocols comprised the execution of either a half-squat (Protocol A) or a lunge exercise (Protocol B), both employing a conical pulley (Versa-Pulley, Costa Mesa, CA, USA). It consisted of 3 sets of 8 repetitions, with the first two increasing flywheel inertia (6 effective repetitions were performed), with 0.048 kg*m^2^ of inertia [10,19] and with 2 min of passive recovery between sets [27]. The alternating lunges exercise (protocol B) used a conical pulley consisting of 3 sets of 10 repetitions (4 effective repetitions per leg, with one for each leg to increase flywheel inertia) using 0.048 kg·m^2^ of inertia [10]. In both protocols, the rope was rewinding at the largest diameter part of the cone [28], with 2′ of passive recovery. The quality of each movement was evaluated by an experienced researcher, who offered feedback to the participants. The depth of the eccentric phase was near 90° knee flexion during the half-squat exercise, and the concentric phase was executed at the maximum speed intended. After each PAPE protocol (half-squat or lunge protocol) and following some of the time intervals proposed by Fu et al. [20] to study the relationship between fatigue and PAPE effects, there were two (first testing), eight (second testing), and twelve minutes (third testing) of break.

### 2.5. Statistical Analyses

JASP v0.19.3 (JASP Team, University of Amsterdam, Amsterdam, The Netherlands) software was used for statistical analyses. Mean and standard deviation (SD) were used to present the data. Before starting the analysis, normality and sphericity assumptions were checked. Normality requires that the dependent variable be approximately normally distributed, while sphericity requires that the variances of the differences between all combinations of factor levels should be the same. The test–retest reliability was assessed using a fixed-effect model. The 2-ways-repeated measures ANOVA was used to evaluate the PAPE effects in every period of time (2, 8, and 12 min) and for both exercises (half-squat or lunge), and the *p*-value for significant difference was set at 0.05. If necessary, the Bonferroni post hoc test was carried out for pairwise comparisons only when significant comparisons (time × exercise) were detected.

Individual data analysis was conducted using the smallest worthwhile change (SWC), calculated as 0.2 × the between-subject standard deviation, to determine meaningful individual responses and to classify athletes as responders or non-responders. Effect size (ES) was interpreted using the Cohen criteria: trivial < 0.2; 0.2 ≤ small < 0.6; 0.6 ≤ moderate < 1.2; 1.2 ≤ large < 2.0; and very large ≥ 2.0. Figures display mean values with 95% confidence intervals calculated from the standard error of the mean (SEM). Minor technical variability inherent to field-based measurements was considered during data screening, and one participant was excluded from the final analyses due to incomplete or invalid data.

## 3. Results

Descriptive values (mean ± SD) for CMJ, triple hop, and T-505 performances across time points and protocols are shown in Table 1. These results provide a general overview of performance trends before the inferential analyses.

**Figure 3:**
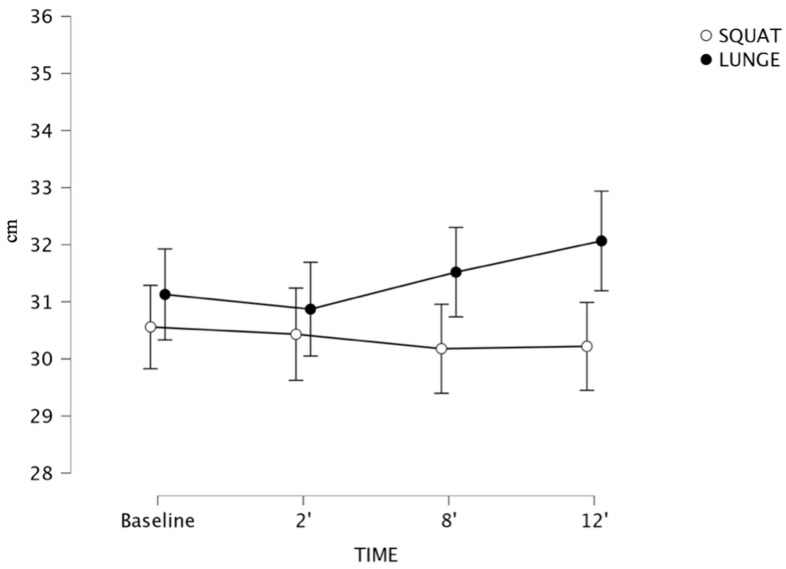
CMJ performance following flywheel half-squat (white) compared to flywheel lunge (black) post-activation performance enhancement (PAPE) conditions. Error bars represent 95% confidence intervals (*n* = 21).

There were significant within-subject differences across the three protocols (*p* < 0.01), but no significant interactions were observed for time or exercise. Specifically, for the CMJ (see Figure 3) [SQ: F (1, 20) = 0.975, *p* = 0.411; LG: F (1, 20) = 0.533, *p* = 0.661] performance, there was no significance for time and exercises performed. No other post hoc comparisons reported meaningful differences.

**Figure 4:**
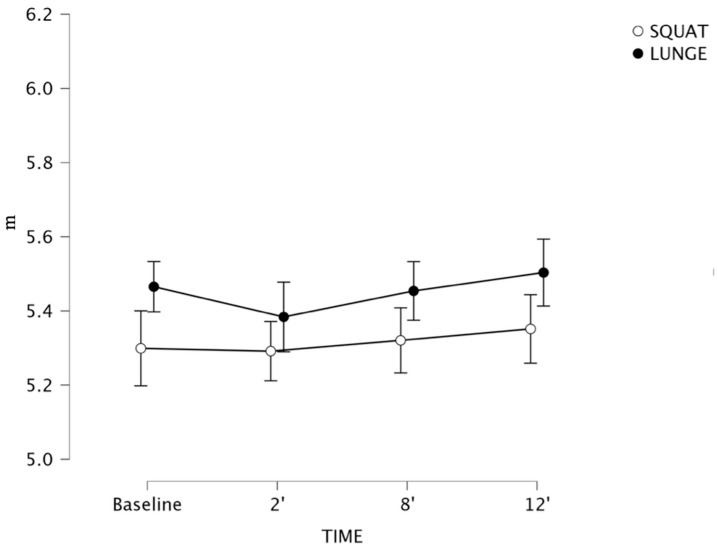
Triple-hop performance following flywheel half-squat (white) compared to flywheel lunge (black) post-activation performance enhancement (PAPE) conditions. Error bars represent 95% confidence intervals (*n* = 21).

For the interaction between triple-hop (see Figure 4) [SQ: F (1, 20) = 1.878, *p* = 0.144; LG: F (1, 20) = 2.148, *p* = 0.105] performances, there was no significance for time and exercises performed. No other post hoc comparisons reported meaningful differences.

**Figure 5:**
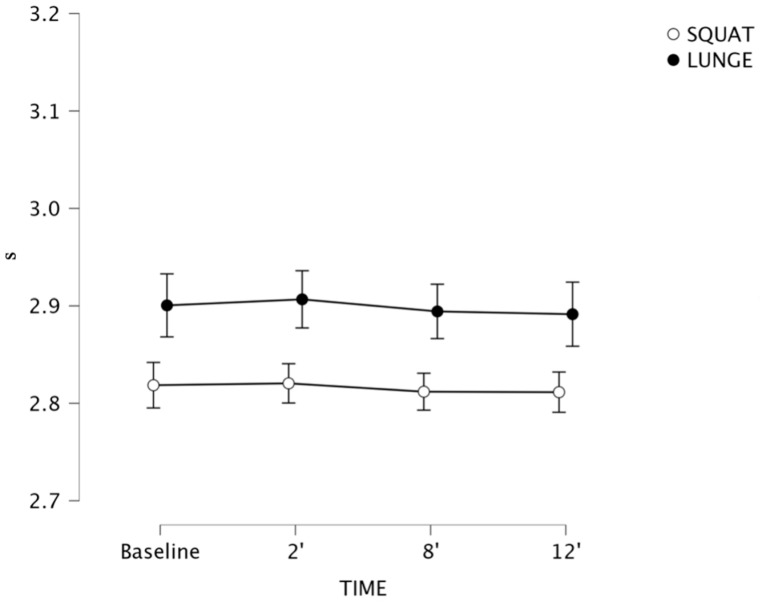
T-505 performance following flywheel half-squat (white) compared to flywheel lunge (black) post-activation performance enhancement (PAPE) conditions. Error bars represent 95% confidence intervals (*n* = 21).

For the interaction between T-505 (see Figure 5) [SQ: F (1, 20) = 1.602, *p* = 0.200; LG: F (1, 20) = 0.720, *p* = 0.174] performances, there was no significance for time and exercises performed. No other post hoc comparisons reported meaningful differences.

Due to the small sample size, to confirm the results, each individual target was contrasted with an individual analysis using SWC (see Table 2). Based on this criterion, the proportion of responders and non-responders was calculated for each performance test and conditioning protocol. In the half-squat protocol, 11 out of 21 athletes (52.4%) were classified as global responders in the countermovement jump (CMJ) test, 13 (61.9%) in the triple-hop test, and 13 (61.9%) in the T-505 change-of-direction test. Under the lunge protocol, 10 athletes (47.6%) responded in the CMJ test, 13 (61.9%) in the triple hop, and 11 (52.4%) in the T-505 test. Overall, the distribution of global responders was relatively similar between protocols for each respective test.

## 4. Discussion

The aim of this study was to compare the effects of two different flywheel PAPE protocols (half-squat and lunge exercises) on vertical and horizontal jump performance, as well as change-of-direction ability in female amateur footballers. The main findings of this research are: (1) There were no significant PAPE effects on the female population with < 1.22 RM/BW ratio; (2) individual PAPE responses were found in both protocols between subjects in some of the time windows measured (2, 8, and 12 min); and (3) in addition, no relationship was found between the specificity of PAPE protocols and sport-specific tasks.

Previous research [9,20] reported significant improvements in CMJ after using the flywheel squat exercise as a PAPE protocol. These findings are not in line with the results of the present study, where improvements in CMJ were not found with any of the PAPE protocols, with approximately half (48–52%) of the subjects having PAPE effects in our study. Differences in methodological factors, such as the inertial load applied (e.g., 0.048–0.122 kg·m^2^), the duration of rest intervals (ranging from 2 to 8 min), and participants’ relative strength levels, reported in previous research may partly explain these discrepancies, since each of these variables can modulate the balance between fatigue and potentiation. Much research on the PAPE topic has been performed with the male population. In addition to the results, previous literature applied to the female population [1,29,30] did not show CMJ improvements between two and ten minutes after PAPE protocol based on the flywheel squat exercise. The main reason given by the authors to explain these results was that two minutes appeared to be too short a recovery time to dissipate fatigue and promote potentiation [18]. Regarding the directionality of the PAPE effect, our hypothesis was not confirmed: horizontal PAPE protocol did not show greater improvements than vertical protocol in triple-hop and modified T-505 performances, with the same number of responders in both protocols. According to our results, previous research [9] showed no improvements in standing broad jump and modified T-505 six minutes after vertical activities (i.e., flywheel squat). Related to our study, Cuenca-Fernández et al. [31] studied the influence of vertical-directed lunge exercises in some vertical and horizontal swimming performance parameters. Consistent with our results, these authors found no differences after applying the PAPE protocol in any of the horizontal performance variables analyzed, but in our study, around 62% of the athletes were classified as responders in the triple-hop test and between 52–62% in the T-505, showing moderate and relatively balanced responses across tests. In contrast to our findings, this experiment did observe differences in some of the vertical performance variables studied following the application of the PAPE protocol. Although the T-505 presented moderate responder rates, the absence of significant group-level differences can likely be attributed to the relatively low reliability of this test (ICC = 0.48) and the small absolute magnitude of individual changes, which, while meaningful at the individual level, were insufficient to yield statistically significant group effects. More recently, Fu et al. [20] founded improvements in horizontal activity (such as 10 and 30 m sprints) after flywheel squat protocol after 4 and 8 min using 0.122 kg·m^2^ of inertia. However, the inclusion criteria for this study were harder than in our study (more than three years of experience in strength training and more than 1.5 of RM/BW ratio). Considering the results, the absence of PAPE benefits in horizontal parameters after vertical PAPE protocol can be due to facts like stability characteristics and the difference between the force–velocity relationship in horizontal tasks. Because of this, these factors must be considered when prescribing the variables and type of exercise for the different horizontal activities. However, to the author’s knowledge, there are no flywheel PAPE protocols with one-leg horizontal-directed exercises (i.e., lunge). Secondly, it has been reported that an athlete’s strength level is a crucial factor for PAPE effects [30,31]. According to the criteria by Meier et al. [22] (1 RM/BW = 1.40), athletes were not expert in squat exercise in this study. Low levels of relative strength may explain the absence of PAPE effects, due to the difficulty in dissipating fatigue at different time points. The results of the present study can be explained by the low levels of relative strength of the sample and by the insufficient body temperature and the lack of optimization of contractile properties induced by the warm-up protocol implemented in this study [32]. This factor can modify the relationship in time between fatigue, potentiation, and performance described in classic studies [33].

In addition, previous research on the topic has been performed with males, and more research with females is needed to conclude the gender differences of some of PAPE’s positive effects. A possible explanation of these differences is that males have a higher motor unit firing frequency, more type II fibers, and more cross-sectional area [34]. In contrast, females have a lower number of type II fibers and different muscle responses due to lower levels of strength [35]. This fact may explain why eccentric actions cannot be an optimal way to generate PAPE effects in the female population. As a consequence of all these determinants of the effects of PAPE, it is likely that strength and conditioning specialists must individualize the protocols according to the individual characteristics of the athletes, following the deterministic model proposed by Suchomel et al. [36]. Further, PAPE protocols may be personalized and monitored to adjust the protocol training variables and individual time points [37,38].

### Limitations and Future Directions

This study is not without limitations. First, the athletes’ strength levels were relatively low (i.e., 1.22 RM/BW), which could have influenced our results. It may be possible that athletes with higher levels of strength could have been more responsive to this PAPE protocol. Second, although a familiarization procedure was performed, a longer familiarization time could have allowed the participants to be more responsive to this PAPE protocol. A recent consensus statement on flywheel resistance exercise highlights the importance of familiarization for the enhancement of mechanical outputs [10]. Third, the modified T-505 showed relatively low reliability (ICC = 0.48), which suggests caution when interpreting conclusions derived from this measure, particularly in the classification of responders. Potential small measurement or technical errors could have contributed to variability at the individual level. Finally, in this study, the load for PAPE protocols was not individualized with the inertial load eliciting the athlete’s highest power values, as previously performed by other authors [10]. However, other studies [10,19] employed the most suitable moment of inertia to generate the greater power output. The use of the most appropriate inertial load may be more suitable to obtain a PAPE response.

## 5. Conclusions

Although no significant group-level effects were detected, several athletes showed meaningful individual improvements following both flywheel protocols. These findings suggest that PAPE responses are highly individualized and depend on factors such as training status, test reliability, and the characteristics of the conditioning activity. Therefore, coaches should avoid generalized prescriptions and instead monitor each athlete’s response to optimize the practical application of flywheel-based PAPE protocols.

## 6. Practical Applications

In line with this, strength levels are a crucial factor to beneficiate from PAPE effects. In our sample, once athletes reached a relative strength ratio above 1.2, between 70% and 90% of them were classified as responders. Strength and conditioning coaches should individualize PAPE strategies according to the strength level. Furthermore, the effects of PAPE are not specific to exercises performed. Therefore, strength and conditioning coaches should select the exercises that provide the greatest benefits to their athletes, based on the demands of their sport, while also individualizing the loads used in PAPE protocols.

## Figures and Tables

**Table 1:** Descriptive values (mean ± SD) for CMJ, triple hop, and T-505 performances under squat (SQ) and lunge (LG) protocols at 2′, 8′, and 12′ post-activation.

Protocol	Test	2′ (Mean ± SD)	8′ (Mean ± SD)	12′ (Mean ± SD)
SQ	CMJ	31.00 ± 3.74	30.66 ± 3.71	30.79 ± 3.80
	Triple Hop	5.28 ± 0.37	5.30 ± 0.40	5.32 ± 0.43
	T-505	2.90 ± 0.12	2.87 ± 0.10	2.87 ± 0.09
	CMJ	31.73 ± 3.86	32.07 ± 3.76	32.90 ± 4.20
LG	Triple Hop	5.38 ± 0.42	5.45 ± 0.36	5.47 ± 0.43
	T-505	3.00 ± 0.13	2.97 ± 0.13	2.97 ± 0.13

Note: Values are presented as mean ± standard deviation.

**Table 2:** Number and percentage of responders (R) and non-responders (NR) at each post-activation time point, according to the SWC.

Protocol (*n* = 21)	Test	2′	8′	12′
VERTICAL—BILATERAL	CMJ R	6 (28.6%)	7 (33.3%)	6 (28.6%)
	CMJ NR	15 (71.4%)	14 (66.7%)	15 (71.4%)
	TRIPLE HOP R	8 (38.1%)	8 (38.1%)	11 (52.4%)
	TRIPLE HOP NR	13 (61.9%)	13 (61.9%)	10 (47.6%)
	T-505 R	7 (33.3%)	11 (52.4%)	7 (33.3%)
	T-505 NR	14 (66.7%)	10 (47.6%)	14 (66.7%)
HORIZONTAL—UNILATERAL	CMJ R	5 (23.8%)	9 (42.9%)	9 (42.9%)
	CMJ NR	16 (76.2%)	12 (57.1%)	12 (57.1%)
	TRIPLE HOP R	6 (28.6%)	8 (38.1%)	9 (42.9%)
	TRIPLE HOP NR	15 (71.4%)	13 (61.9%)	12 (57.1%)
	T-505 R	5 (23.8%)	8 (38.1%)	10 (47.6%)
	T-505 NR	16 (76.2%)	13 (61.9%)	11 (52.4%)

Note. R = responders; NR = non-responders; CMJ = countermovement jump; triple hop = horizontal triple hop; modified T-505 = change-of-direction test. The values show the number of time-specific responders/non-responders (R/NR) and the percentage of responders and non-responders (%) at 2′, 8′, and 12′. Global responders are reported in the main text.

## Data Availability

Data are available upon request from the corresponding author.
